# Comparison of plasma metabolic profiling between children with hypertrophic obstructive cardiomyopathy and healthy controls

**DOI:** 10.1097/XCE.0000000000000328

**Published:** 2025-04-25

**Authors:** Shuo Dong, Chuhao Du, Hao Cui, Jie Dong, Shun Liu, Haitao Xu, Yangxue Sun, Mengxuan Zou, Jiangping Song, Jun Yan

**Affiliations:** a Department of Pediatric Cardiac Surgery, National Center for Cardiovascular Disease and Fuwai Hospital, Chinese Academy of Medical Sciences, Peking Union Medical College; b State Key Laboratory of Cardiovascular Disease, Fuwai Hospital, National Center for Cardiovascular Diseases, Chinese Academy of Medical Sciences and Peking Union Medical College, Beijing, China

**Keywords:** cardiovascular disease, children, hypertrophic obstructive cardiomyopathy, liquid chromatography system, plasma metabolomics

## Abstract

**Background:**

The diagnostic value of serum metabolomics in congenital heart disease has been proven. The difference of serum metabolomics between children with hypertrophic obstructive cardiomyopathy (HOCM) and normal children is unknown.

**Methods:**

Fasting blood samples of 24 symptomatic children with HOCM, 11 children with left ventricular outflow tract obstruction because of other cardiac anomalies (non-HOCM group) and 41 normal controls were obtained. The targeted metabolomic approach was performed using a Vanquish ultra-performance liquid chromatography system coupled to a Q-Exactive HF mass spectrometer.

**Results:**

The plasma level of 79 out of 224 metabolites were significantly changed (|log2FC| > 1 and *P*_adj._ < 0.05) between the HOCM and the normal group. There was no significant difference between the HOCM and non-HOCM groups. A total of 79 significantly changed metabolites between the HOCM and the normal group were significantly enriched in two pathways, including purine metabolism (*P* < 0.001) and thiamine metabolism (*P* = 0.034).

**Conclusion:**

Our results identified the significant changes of plasma metabolite between children with HOCM and healthy children, which is helpful for understanding the pathogenesis of HOCM and early diagnosis.

**Key points**

- Differential metabolites of plasma can help to better understand the pathogenesis of diseases and provide potential diagnostic value.
- This study reveals for the first time the significant changes in blood metabolism between children with hypertrophic obstructive cardiomyopathy and healthy controls.
- A total of 79 metabolites were significantly different between hypertrophic obstructive cardiomyopathy and the normal group.
- Purine and thiamine metabolic pathways are the main differential pathways between children with hypertrophic obstructive cardiomyopathy and healthy controls.

## Introduction

Hypertrophic cardiomyopathy (HCM) is the most common hereditary cardiomyopathy with an incidence of one out of 500 in the population [1]. Compared with adult-onset HCM, childhood-onset carry a higher risk of life-threatening ventricular arrythmias, and have a greater need for advanced heart failure (HF) therapies [2]. So it is of great significance for the early diagnosis and treatment of HCM in children. The diagnostic value of serum metabolomics in congenital heart disease (CHD) has been proven [3], but there is still a lack of metabolomics studies in children with hypertrophic obstructive cardiomyopathy (HOCM). Because HOCM lacks a clear genotype–phenotype relationship [2], early identification of mutation carriers who are prone to developing phenotype-positive HOCM is difficult [4,5]. Plasma metabolomic testing may help achieve this goal. In addition, imaging studies have limitations in the differential diagnosis of HCM and other disorders that can cause ventricular hypertrophy (aortic stenosis, coarctation of the aorta, etc.).

This study aimed to identify the differential metabolites of HOCM in children by comparing the blood metabolomics of children with HOCM, normal children, and children with non-HOCM left ventricular outflow tract stenosis, which will help to better understand the pathogenesis of this disease and provide early diagnostic and differential diagnostic value.

## Methods

### Study design

In this cross-sectional study, 75 subjects were selected from the Fuwai Hospital including 24 patients with HOCM (5.15 ± 3.58 years old), 11 patients with left ventricular outflow tract obstruction (non-HOCM group, 2.51 ± 2.43 years old) because of other cardiac anomalies (including aortic stenosis and aortic coarctation) and 41 normal controls (5.81 ± 4.12 years old). No cardiac diseases were found in all healthy controls that were confirmed by clinical screening and echocardiography. Fasting blood samples were obtained from all subjects before undergoing surgery and immediately centrifuged at 4000 rpm for 10 min, then the supernatant plasma was collected and stored at −80 °C. This study was approved by the Ethics Committee of the Fuwai Hospital and was conducted in accordance with the 1964 Declaration of Helsinki. Written informed consent was obtained from all subjects included in this study. The basic characteristics of subjects were provided in Table 1.

**Table 1:** Clinical features.

Variables	HOCM (*n* = 24)	Non-HOCM LVOTO (*n* = 11)	*P* value
Age at surgery (years)	5.15 ± 3.58	2.51 ± 2.43	0.034
Male	50.0%	72.7%	0.281
Family history (HCM/SCD)	12.5%	0	0.536
Maximal gradients of LVOT (mmHg)	75.54 ± 32.48	51.98 ± 28.85	0.008
Maximal septal thickness (mm)	16.77 ± 4.91	5.91 ± 1.30	<0.001
Posterior wall thickness of LV (mm)	9.01 ± 3.55	5.55 ± 1.22	0.004
Symmetrical hypertrophy	12.5%	9.15%	1.000
Midventricular obstruction	37.5%	0	0.033
Anomalous SMVA	62.5	0	0.001
LVEDD (mm)	29.42 ± 7.76	27.18 ± 6.32	0.410
LAD (mm)	28.88 ± 9.60	18.91 ± 4.85	<0.001
Mean MR grade	2.29 ± 1.46	0.36 ± 0.67	<0.001
None	3	8	
Trivial/mild	6	2	
moderate	3	1	
Moderately severe	5	0	
severe	7	0	
SAM	87.5%	9.1%	<0.001
Ejection fractions (%)	74.56 ± 7.27	67.00 ± 6.97	0.007
Residual obstruction (%)	20.8%	9.1%	0.640
Recurrent obstruction (%)	12.5%	9.1%	1.000
Death	4.2%	0	1.000

HCM, hypertophic cardiomyopathy; HOCM, hypertrophic obstructive cardiomyopathy; LAD, left atrium dimension; LV, left ventricle; LVEDD, left ventricular end-diastolic dimension; LVOT, left ventricular outflow tract; MR, mitral regurgitation; SAM, systolic anterior motion; SCD, sudden cardiac death; SMVA, subvalvular mitral valve apparatus.

### Metabolite extraction from plasma samples

The plasma metabolite extraction was previously described. In brief, 50 μl aliquot of plasma was mixed with 450 μl extract liquor then vortexing for 5 min. Next, the mixture was centrifuged at 18 400 *g* at 4 °C for 30 min. After centrifugation, the supernatant was collected and transferred to a 2 ml brown vial for liquid chromatography-tandem mass spectrometry (LC-MS/MS) analysis. The quality control sample was produced by the mixture composed of equal volume from each plasma sample.

### LC-MS/MS analysis

The targeted metabolomic approach was performed using a Vanquish ultra-performance liquid chromatography system coupled to a Q-Exactive HF mass spectrometer (Thermo Fisher Scientific, Seattle, Washington, USA). A modified method was used for metabolite separation [6]. The ACQUITY BEH amide column (50 × 2.1 mm, 1.7 μm; Waters) and BEH HILIC column (50 × 2.1 mm, 1.7 μm; Waters, Milford, Massachusetts, USA) were used for electrospray ionization (ESI)+ and ESI− mode, respectively. Solvent A was H_2_O containing 10 mM ammonium formate and solvent B was acetonitrile. For the BEH amide column, the gradient elution was as follows: 0–1.0 min, 95% B; 1.0–7.0 min, 95–70% B; 7.0–10.0 min, 70–30% B; 10.0–12.5 min, 30% B; 12.5–13.0 min, 95% B; 13.0–15.0 min, 95% B. The flow rate was set at 0.4 ml/min and column temperature at 45 °C. For the BEH HILIC column, the gradient elution was as follows: 0–1.0 min, 95% B; 1.0–6.0 min, 95–90% B; 6.0–10.5 min, 90–85% B; 10.5–12.5 min, 85–70% B; 12.5–14.5 min, 70% B; 14.5–15.0 min, 70–95% B; 15.0–18.0 min, 95% B (Table S1, Supplemental Digital Content 1, http://links.lww.com/CAEN/A68). The flow rate was set at 0.4 ml/min and column temperature at 25 °C. The data acquisition was performed in the parallel reaction monitoring mode. The MS parameters were set as follows: resolution 15 000, new chemical entity 20, 40, 60 eV, spray voltage 3.5 kV for ESI+ and 4.0 kV for ESI−, capillary temperature set at 320 °C, sheath gas set at 25, and aux gas set at 5. The concentration of each metabolite was calculated by area response from an independent standard curve.

### Data processing

The raw data were processed using Trace Finder (version 4.1; Thermo Fisher Scientific) for peak picking and feature alignment. The precursor and fragment ions from MS1 and MS2 were utilized for identification and quantification. The concentration of metabolites was calculated according to an independently external standard curve. Orthogonal partial least squares-discriminant analysis (OPLS-DA) was performed by SIMCA version 15.0 software (Umetrics, Umeå, Sweden). The Kyoto Encyclopedia of Genes and Genomes pathway enrichment analysis and Pearson correlation analysis were performed on Metaboanalyst 5.0 (https://www.metaboanalyst.ca/). The receiver operating characteristic (ROC) analysis was applied with SPSS 20.0 (IBM Corp., Armonk, New York, USA). The cutoff threshold is the metabolite concentration at the peak of the Youden index.

### Statistical analysis

Differences in metabolite levels between HOCM, non-HOCM and normal were assessed with Mann–Whitney *U* tests that were performed by SPSS 20.0 (IBM Corp.). All data were expressed as the median with the interquartile range. *P* values adjusted by a false discovery rate less than 0.05 were considered to indicate statistical significance.

## Results

### Clinical features

See Table 1.

### General description and multivariate analysis of plasma metabolomics data for hypertrophic obstructive cardiomyopathy, normal, and nonhypertrophic obstructive cardiomyopathy group

#### Hypertrophic obstructive cardiomyopathy vs. normal

**Fig. 1:**
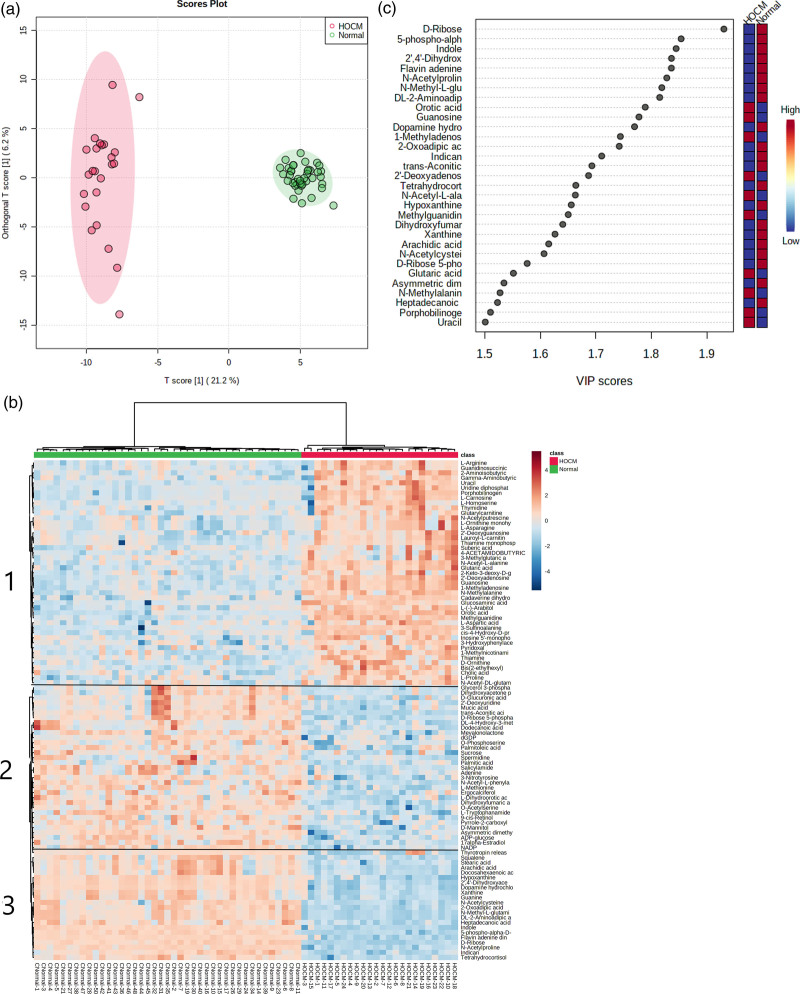
Separation of plasma metabolic profiles between HOCM and normal group. (a) OPLS-DA score plot of plasma metabolites. (b) Hierarchical clustering analysis of plasma metabolites between two groups. (c) Bubble plot of VIP value. HOCM, hypertrophic obstructive cardiomyopathy; OPLS-DA, orthogonal partial least squares-discriminant analysis; VIP, variable importance in projection.

**Fig. 2:**
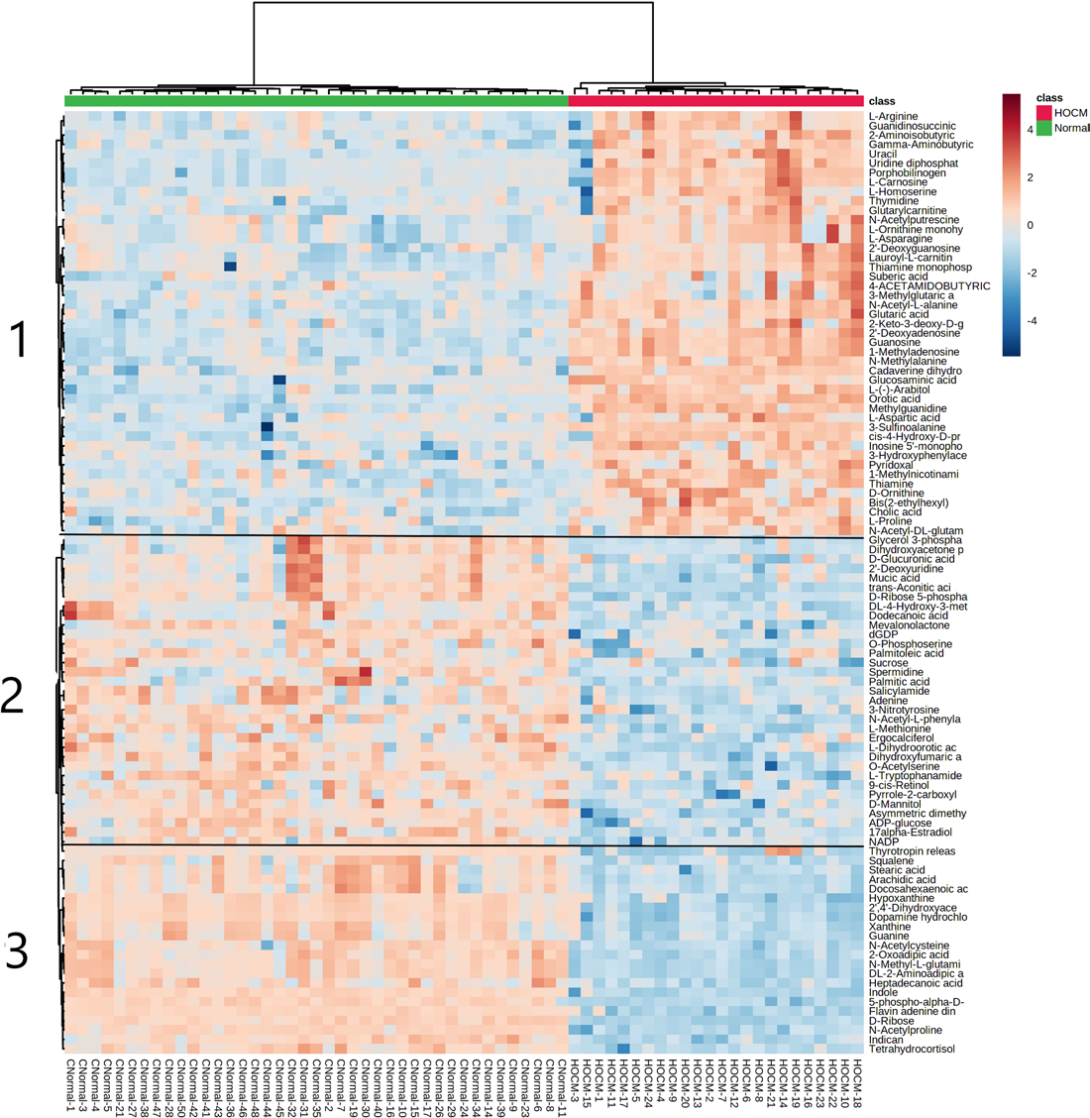
Hierarchical clustering analysis of plasma metabolites between two groups. HOCM, hypertrophic obstructive cardiomyopathy.

Our metabolomics methods can measure 308 plasma metabolites that are confirmed by retention time, precursor, and fragment ions acquired from our authenticated standard database. Among these, 224 metabolites were detected in at least 70% of plasma samples. The OPLS-DA score plot revealed that a large number of plasma levels of metabolites were changed between the HOCM and normal groups (Fig. 1a). The results showed that the plasma level of 79 out of 224 metabolites were significantly changed (|log2FC| > 1 and *P*_adj._ < 0.05) between two groups. To further investigate the difference of plasma metabolome between the two groups, we assembled metabolites with similar characteristics using an unsupervised hierarchical clustering method (Fig. 2). A total of three metabolite clusters were divided and their metabolite expression characteristics were shown in Fig. 1b. Several substrates associated with aerobic respiration such as fatty acids (arachidic acid and stearic acid) and purine metabolites (guanine and xanthine) were assigned into the cluster 3 and were remarkably decreased in HOCM compared with normal group. Many amino acids (L-arginine, 2-aminoisobutyric, and 3-sulfinoalanine) were assigned into cluster 1 and were increased in the HOCM group compared with the normal group.

A total of 31 metabolites showed variable importance in projection (VIP) greater than 1.5 that were considered important for discrimination (Fig. 1c). The VIP value of the OPLS-DA model were shown in Table S1, Supplemental Digital Content 1, http://links.lww.com/CAEN/A68.

#### Hypertrophic obstructive cardiomyopathy vs. nonhypertrophic obstructive cardiomyopathy

**Fig. 3:**
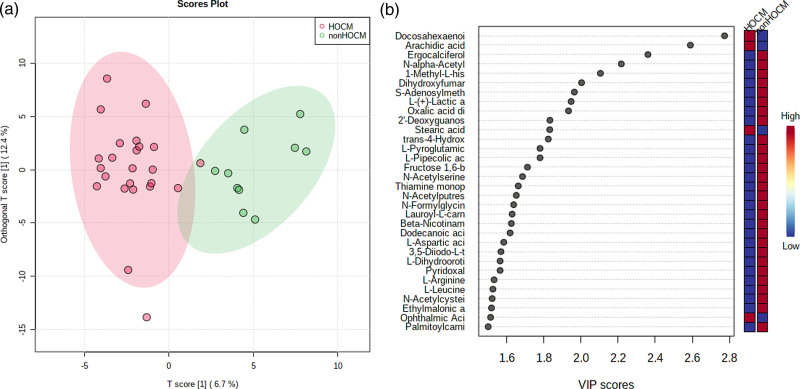
Separation of plasma metabolic profiles between HOCM and non-HOCM groups. (a) OPLS-DA score plot of plasma metabolites. (b) Bubble plot of VIP value. HOCM, hypertrophic obstructive cardiomyopathy; VIP, variable importance in projection.

The OPLS-DA score plot revealed that a large number of plasma levels of metabolites were changed between the HOCM and non-HOCM groups (Fig. 3a). To further investigate the difference of plasma metabolome between the two groups, we assembled metabolites with similar characteristics using an unsupervised hierarchical clustering method which showed no significant difference.

A total of 12 metabolites showed a VIP greater than 1.8 that were considered important for discrimination (Fig. 3b). The VIP value of the OPLS-DA model were shown in Table S2, Supplemental Digital Content 1, http://links.lww.com/CAEN/A68.

### Pathway analysis for hypertrophic obstructive cardiomyopathy and normal group

**Fig. 4:**
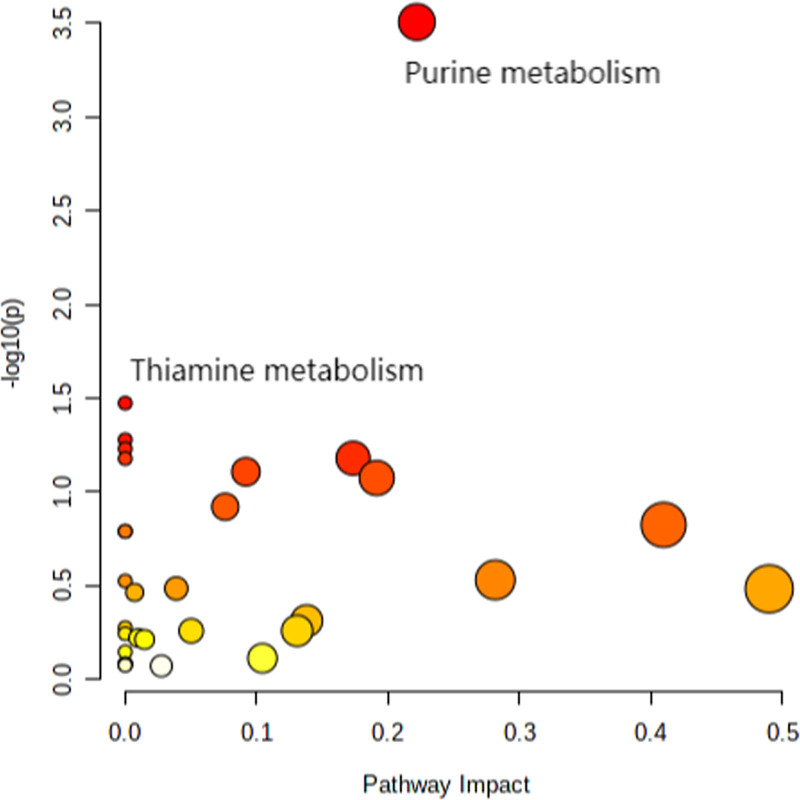
Pathway analysis of 32 significantly changed plasma metabolites between HOCM and normal group screened by fold change and *P*_adj._ value. HOCM, hypertrophic obstructive cardiomyopathy.

Pathway analysis of the significantly changed metabolites were conducted. A total of 79 metabolites were significantly enriched in two pathways, including purine metabolism (*P* < 0.001) and thiamine metabolism (*P* = 0.034) (Fig. 4).

### Identification of differentially expressed metabolites between hypertrophic obstructive cardiomyopathy and normal groups

**Fig. 5:**
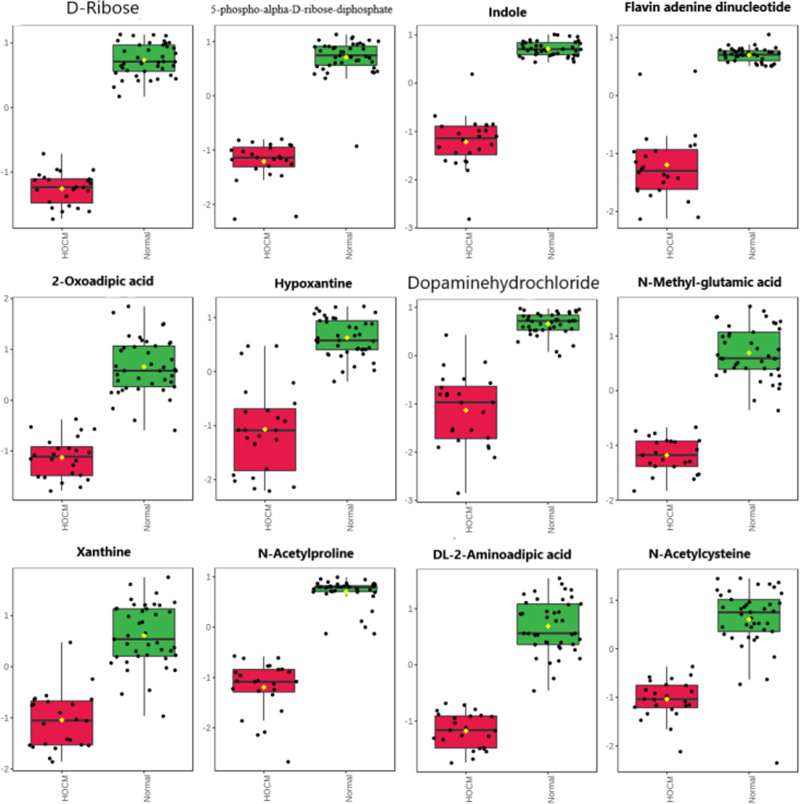
Twelve typical DEMs between HOCM and normal group. The plasma concentration of all metabolites was significantly different between the HOCM and the normal group (*P*_adj._ < 0.001). The *y*-axis represents the normalized plasma concentration. DEM, differentially expressed metabolites; HOCM, hypertrophic obstructive cardiomyopathy.

We defined the differentially expressed metabolites (DEMs) between HOCM and normal groups according to the following rules: (Fig. 5) (a) |log_2_FC| > 1 and *P*_adj._ < 0.05, (b) VIP > 1.5, (c) |*p* (corr)| > 0.8 and |*p*[1]| > 2. A total of 18 metabolites were screened as DEMs (Table S3, Supplemental Digital Content 1, http://links.lww.com/CAEN/A68). Typical DEMs are shown in Fig. 4. D-ribose (log_2_FC = −1.44, *P*_adj._ = 9.41 × 10^−40^), 5-phospho-alpha-D-ribose 1-diphosphate (log_2_FC = −1.61, *P*_adj._ = 3.62 × 10^−30^), indole (log_2_FC = −3.40, *P*_adj._ = 1.21 × 10^−30^), 2′,4′-dihydroxyacetophenone (log_2_FC = −3.60, *P*_adj._ = 8.16 × 10^−26^), dopamine hydrochloride (log_2_FC = −3.19, *P*_adj._ = 2.60 × 10^−21^), 2-oxoadipic acid (log_2_FC = −1.39, *P*_adj._ = 6.38 × 10^−21^) were significantly decreased in HOCM group. Many metabolites of purine were significantly decreased in the HOCM group, including flavin adenine dinucleotide (FAD) (log_2_FC = −2.86, *P*_adj._ = 1.31 × 10^−27^), hypoxanthine (log_2_FC = −2.83, *P*_adj._ = 3.32 × 10^−17^), xanthine (log_2_FC = −3.61, *P*_adj._ = 5.35 × 10^−16^). Furthermore, several metabolites of amido acid catabolism were also significantly decreased in HOCM group, including indican (log_2_FC = −2.43, *P*_adj._ = 1.23 × 10^−20^), *N*-methyl-L-glutamic acid (log_2_FC = −1.53, *P*_adj._ = 1.23 × 10^−25^), DL-2-aminoadipic acid (log_2_FC = −1.50, *P*_adj._ = 3.28 × 10^−25^), *N*-acetylcysteine (log_2_FC = −1.65, *P*_adj._ = 1.31 × 10^−15^), and *N*-acetylproline (log_2_FC = −2.06, *P*_adj._ = 7.91 × 10^−28^).

### Analysis of biomarkers

**Fig. 6:**
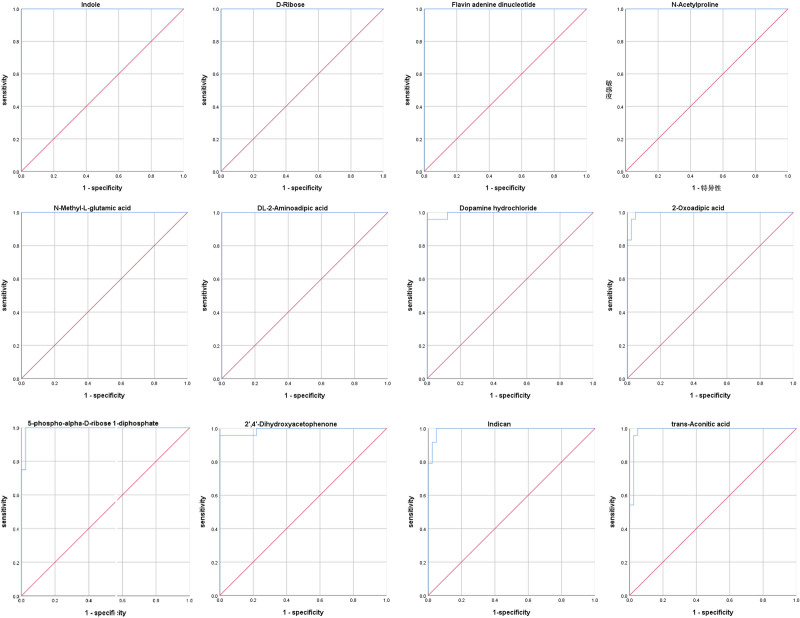
The predictive effect of potential biomarkers was evaluated by ROC curves in discriminating patients with HOCM from healthy children. HOCM, hypertrophic obstructive cardiomyopathy; ROC, receiver operating characteristic.

ROC analysis was used to evaluate the diagnostic value of typical DEMs in discriminating children with HOCM from healthy children (Fig. 6). The area under the curve (AUC) of 12 typical DEMs was shown in Table S4, Supplemental Digital Content 1, http://links.lww.com/CAEN/A68. The cutoff threshold of D-ribose, indole, FAD, *N*-acetylproline, *N*-methyl-L-glutamic acid, and DL-2-aminoadipic acid were 436.9445, 4914.0265, 236.3065, 74.4960, 45242.2750, and 841.1190 μg/L to generate a maximum diagnostic efficiency with AUC = 1. In addition, positive and negative predictive values for these metabolites at cutoff threshold were all 100%.

## Discussion

Many studies have demonstrated the value of plasma metabolome analysis for uncovering molecular mechanisms of CHD pathogenesis and identifying biomarkers for diagnostic and therapeutic purposes. In this study, we first carry out an absolutely quantitative metabolomics based on ultra performance liquid chromatography-discriminant analysis to describe the differentially metabolic profiling between HOCM, non-HOCM, and normal groups. Our results showed disease-specific metabolic signatures and indicated many DEMs involved in purine and thiamine metabolism between HOCM and normal groups. In the 12 identified potential biomarkers in plasma, a maximum diagnostic efficiency of D-ribose, indole, FAD, *N*-acetylproline, *N*-methyl-L-glutamic acid, DL-2-aminoadipic acid was observed with AUC = 1.

Through quantitative metabolomic analysis, we identified 35 significantly upregulated and 44 downregulated metabolites from a total of 224 metabolites. Pathway enrichment analysis showed that the significantly changed metabolites enriched on purine metabolism and thiamine metabolism.

The pathophysiological mechanisms of HCM include decreased coronary density [7–9], cardiac hypertrophy, myocardial fibrosis, diastolic dysfunction, and so forth [10]. The reduction of various purine metabolites in plasma shown in this study may be related to each of these mechanisms. Purine nucleotides are synthesized by both de-novo and salvage pathways. The de-novo pathways create these phosphorylated ring structures from simple precursors, such as CO_2_, glycine, and glutamine; the salvage pathways reutilize of purine and bases [11,12].

Purine nucleosides and nucleotides promote the migration and proliferation of vascular smooth muscle and endothelial cells through P1 and P2Y receptors and play a long-term nutritional role in the process of angiogenesis [13]. The results of this study showed that various purine metabolites in the plasma of patients with HCM were significantly reduced, and the sparse coronary blood vessels in patients with HCM may be related to this [7–9].

HCM involves not only myocardial hypertrophy but also a certain degree of myocardial ischemia [8]. This study showed that serum guanosine [fold change (FC) = 12.444, *P*_adj._ = 1.08 × 10^−21^] was significantly increased in HCM, which is a synthetic substrate of cyclic GMP (cGMP). The cGMP exerts cardioprotective effects against ischemia/reperfusion injury through activation of cyclic GMP–dependent protein kinase [14,15]. In addition, several studies have shown that cGMP inhibits hypertrophy, and regulates contractile function and cardiac remodeling [16].

FAD (FC = 0.138, *P*_adj._ = 1.31 × 10^−27^) can inhibit pathological cardiac hypertrophy and fibrosis through activating short-chain acyl-CoA dehydrogenase [6], which was significantly reduced in this study. Also, adenosine (FC = 1.919, *P*_adj._ = 0.0013) was significantly reduced which, acting via A2B receptors, inhibits collagen and protein synthesis in cardiac fibroblasts [13].

Furthermore, thiamine metabolism was significantly enriched, and thiamine and thiamine monophosphate were found to be significantly upregulated. Previous studies showed that a deficiency in thiamine can lead to neurological abnormalities and congestive HF [17]. This result may indicate thiamine metabolism is concerned in the progression of HOCM.

Overall, there are significant differences in plasma metabolites between HOCM and non-HOCM groups. The further focus of this study is to identify the HCM mutation carriers without myocardial hypertrophy. Disease-specific metabolic signatures and many DEMs between the HOCM and the normal group involved in our results showed that plasma metabolomic testing may help early identification of HCM children.

## Limitation

First, the retrospective nature of the study may reinforce the risk of selection bias. Second, this is a single-center study, so the population with distinctive phenotypes may not be representative for the global picture of pediatric obstructive HCM. Third, the small number of patients and events warrant caution in interpreting our analyses.

### Conclusion

Our results identified the significant differences of plasma metabolite between children with HOCM and healthy children, which is helpful for early diagnosis. Metabolite pathways associated with purine and thiamine in childhood HOCM are distinctive.

## Acknowledgements

This study was supported by the Clinical and Translational Research Fund of the Chinese Academy of Medical Sciences (2022-1727).

The institutional review board (IRB) or equivalent ethics committee of the Fuwai Hospital approved the study protocol and publication of data. The patients provided informed written consent for the publication of the study data.

The datasets generated during the current study are not publicly available due to individual privacy but are available from the corresponding author on reasonable request.

### Conflicts of interest

There are no conflicts of interest.

## Supplementary Material


