# Supplementary material for: Comparison of plasma metabolic profiling between children with hypertrophic obstructive cardiomyopathy and healthy controls
Source: Cardiovasc Endocrinol Metab. 2025 Apr 25;14(2):e00328. doi: 10.1097/XCE.0000000000000328 (PMC12037094; doi:10.1097/XCE.0000000000000328)
Supplement: Supplementary file 1 [file xce-14-e00328-s001.doc]

Table S1. The OPLS-DA parameters of differential expressed metabolites between HOCM and Normal group

| No | Metabolite name | VIP | p(corr) | p[1] |
| --- | --- | --- | --- | --- |
| 1 | D-Ribose | 1.930637281 | 0.9688 | 7.2733 |
| 2 | 5-phospho-alpha-D-ribose 1-diphosphate | 1.853644127 | 0.93016 | 6.9832 |
| 3 | Indole | 1.844626094 | 0.92564 | 6.9492 |
| 4 | 2',4'-Dihydroxyacetophenone | 1.836266559 | 0.92144 | 6.9178 |
| 5 | Flavin adenine dinucleotide | 1.836123589 | 0.92137 | 6.9172 |
| 6 | N-Acetylproline | 1.827809938 | 0.9172 | 6.8859 |
| 7 | N-Methyl-L-glutamic acid | 1.81891048 | 0.91274 | 6.8524 |
| 8 | DL-2-Aminoadipic acid | 1.815064563 | 0.91081 | 6.8379 |
| 9 | Dopamine hydrochloride | 1.769726195 | 0.88805 | 6.6671 |
| 10 | 2-Oxoadipic acid | 1.742211955 | 0.87425 | 6.5634 |
| 11 | Indican | 1.710615377 | 0.85839 | 6.4444 |
| 12 | trans-Aconitic acid | 1.692964018 | 0.84954 | 6.3779 |
| 13 | Tetrahydrocortisol | 1.663983216 | 0.83499 | 6.2687 |
| 14 | Hypoxanthine | 1.655842304 | 0.83091 | 6.238 |
| 15 | Dihydroxyfumaric acid | 1.640532132 | 0.82322 | 6.1804 |
| 16 | Xanthine | 1.626476484 | 0.81617 | 6.1274 |
| 17 | Arachidic acid | 1.615167701 | 0.8105 | 6.0848 |
| 18 | N-Acetylcysteine | 1.606743908 | 0.80627 | 6.0531 |

Table S2. The OPLS-DA parameters of differential expressed metabolites between HOCM and non-HOCM group

| No | Metabolite name | VIP | p(corr) | p[1] |
| --- | --- | --- | --- | --- |
| 1 | Docosahexaenoic acid | 2.773846241 | -0.71914 | -2.6995 |
| 2 | Arachidic acid | 2.589425905 | -0.67132 | -2.5198 |
| 3 | Ergocalciferol | 2.360481693 | 0.61197 | 2.297 |
| 4 | N-alpha-Acetyl-L-lysine | 2.217022894 | 0.57478 | 2.1576 |
| 5 | 1-Methyl-L-histidine | 2.104307689 | 0.54555 | 2.0479 |
| 6 | Dihydroxyfumaric acid | 2.002786358 | 0.51923 | 1.949 |
| 7 | S-Adenosylmethionine | 1.963830303 | 0.50913 | 1.911 |
| 8 | L-(+)-Lactic acid | 1.946290334 | 0.50459 | 1.8938 |
| 9 | Oxalic acid dihydrate | 1.932322701 | 0.50097 | 1.8804 |
| 10 | 2'-Deoxyguanosine 5'-monophosphate | 1.832071753 | 0.47497 | 1.7827 |
| 11 | Stearic acid | 1.83116408 | -0.47474 | -1.7821 |
| 12 | trans-4-Hydroxy-L-proline | 1.823304454 | 0.4727 | 1.7742 |

Table S3. The plasma concentration of differential expressed metabolites

| **No** | **Metabolite name** | **Plasma concentration** | | **Log_2_FC** | **adj.p value** |
| --- | --- | --- | --- | --- | --- |
|  |  | HOCM  (mean±SEM) | Normal (mean±SEM) |  |  |
| 1 | D-Ribose | 262.679±33.26 | 711.135±88.01 | -1.4368 | 9.41E-40 |
| 2 | 5-phospho-alpha-D-ribose 1-diphosphate | 579.231±109.42 | 1765.388±283.51 | -1.6078 | 3.62E-30 |
| 3 | Indole | 803.729±762.07 | 8464.940±1786.92 | -3.3967 | 1.21E-30 |
| 4 | 2',4'-Dihydroxyacetophenone | 117.432±187.39 | 1426.383±555.84 | -3.6025 | 8.16E-26 |
| 5 | Flavin adenine dinucleotide | 43.993±54.65 | 319.683±49.03 | -2.8613 | 1.31E-27 |
| 6 | N-Acetylproline | 40.844±13.56 | 169.910±28.94 | -2.0566 | 7.91E-28 |
| 7 | N-Methyl-L-glutamic acid | 31584.464±5427.87 | 91303.486±23822.93 | -1.5315 | 1.22E-25 |
| 8 | DL-2-Aminoadipic acid | 613.014±104.44 | 1733.448±443.49 | -1.4997 | 3.28E-25 |
| 9 | Dopamine hydrochloride | 148.940±187.68 | 1356.016±416.66 | -3.1866 | 2.60E-21 |
| 10 | 2-Oxoadipic acid | 4260.570±909.98 | 11133.997±3275.58 | -1.3859 | 6.38E-21 |
| 11 | Indican | 236.934±126.46 | 1274.284±612.14 | -2.4271 | 1.23E-20 |
| 12 | trans-Aconitic acid | 193.522±37.26 | 504.171±214.69 | -1.3814 | 4.11E-18 |
| 13 | Tetrahydrocortisol | 723.684±289.27 | 2928.896±1503.81 | -2.0169 | 2.28E-17 |
| 14 | Hypoxanthine | 50.584±72.87 | 358.657±188.47 | -2.8258 | 3.32E-17 |
| 15 | Dihydroxyfumaric acid | 1652.304±423.88 | 3633.218±1042.28 | -1.1368 | 5.15E-17 |
| 16 | Xanthine | 12.063±16.03 | 147.579±127.43 | -3.6128 | 5.35E-16 |
| 17 | Arachidic acid | 846.308±283.35 | 4195.774±2783.53 | -2.3097 | 3.50E-15 |
| 18 | N-Acetylcysteine | 360.259±87.50 | 1128.069±388.71 | -1.6467 | 1.31E-15 |

Table S4. The ROC results of potential biomarkers between HOCM and normal group

| **No** | **Metabolite name** | **AUC** | **95% CI** | **Sensitivity (%)** | **Specificity(%)** | **Threshold** |
| --- | --- | --- | --- | --- | --- | --- |
| 1 | D-Ribose | 1.000 |  | 1.000 | 1.000 | 436.9445 |
| 2 | Indole | 1.000 |  | 1.000 | 1.000 | 4914.0265 |
| 3 | Flavin adenine dinucleotide | 1.000 |  | 1.000 | 1.000 | 236.3065 |
| 4 | N-Acetylproline | 1.000 |  | 1.000 | 1.000 | 74.4960 |
| 5 | N-Methyl-L-glutamic acid | 1.000 |  | 1.000 | 1.000 | 45242.2750 |
| 6 | DL-2-Aminoadipic acid | 1.000 |  | 1.000 | 1.000 | 841.1190 |
| 7 | Dopamine hydrochloride | 0.995 | 0.984-1.000 | 0.958 | 1.000 | 420.8645 |
| 8 | 2-Oxoadipic acid | 0.995 | 0.985-1.000 | 1.000 | 0.951 | 6569.1905 |
| 9 | 5-phospho-alpha-D-ribose 1-diphosphate | 0.994 | 0.981-1.000 | 1.000 | 0.976 | 1052.9585 |
| 10 | Indican | 0.993 | 0.980-1.000 | 1.000 | 0.951 | 584.6935 |
| 11 | 2',4'-Dihydroxyacetophenone | 0.991 | 0.972-1.000 | 0.958 | 1.000 | 368.5245 |
| 12 | trans-Aconitic acid | 0.988 | 0.965-1.000 | 1.000 | 0.951 | 338.3035 |
